# Architecture engineering of nanostructured catalyst via layer-by-layer adornment of multiple nanocatalysts on silica nanorod arrays for hydrogenation of nitroarenes

**DOI:** 10.1038/s41598-021-02312-0

**Published:** 2022-01-06

**Authors:** Kootak Hong, Jun Min Suh, Tae Hyung Lee, Sung Hwan Cho, Seeram Ramakrishna, Rajender S. Varma, Ho Won Jang, Mohammadreza Shokouhimehr

**Affiliations:** 1 Department of Materials Science and Engineering, Research Institute of Advanced Materials, Seoul National University, Seoul, 08826 Republic of Korea; 2 Center for Nanotechnology and Sustainability, Department of Mechanical Engineering, National University of Singapore, Singapore, 119260 Singapore; 3 Regional Centre of Advanced Technologies and Materials, Czech Advanced Technology and Research Institute, Palacký University, Šlechtitelů 27, 783 71 Olomouc, Czech Republic

**Keywords:** Nanoscience and technology, Catalysis

## Abstract

Direct consideration for both, the catalytically active species and the host materials provides highly efficient strategies for the architecture design of nanostructured catalysts. The conventional wet chemical methods have limitations in achieving such unique layer-by-layer design possessing one body framework with many catalyst parts. Herein, an innovative physical method is presented that allows the well-regulated architecture design for an array of functional nanocatalysts as exemplified by layer-by-layer adornment of Pd nanoparticles (NPs) on the highly arrayed silica nanorods. This spatially confined catalyst exhibits excellent efficiency for the hydrogenation of nitroarenes and widely deployed Suzuki cross-coupling reactions; their facile separation from the reaction mixtures is easily accomplished due to the monolithic structure. The generality of this method for the introduction of other metal source has also been demonstrated with Au NPs. This pioneering effort highlights the feasibility of physically controlled architecture design of nanostructured catalysts which may stimulate further studies in the general domain of the heterogeneous catalytic transformations.

## Introduction

Catalysts are sine qua non for most of the chemical processes to produce fine chemicals, pharmaceuticals, petrochemicals, among plethora of other fine chemicals^1–7^. From the industrial point of view, heterogeneous catalysts are often preferred to their homogeneous counterparts because of low cost, easy separation from reaction mixture, stability, and reusability^8–10^. In addition, the use of heterogeneous catalysts in continuous flow processes have several advantageous features such as easy mass production, automation, higher yields, and quality of products^11–14^.

Despite these remarkable advantages, one of the critical issues in heterogeneous catalysts is their low catalytic activity and selectivity^15–17^. In particular, very low catalytic activity and the rapid decay in efficiency are inevitable when they are stabilized in the non-porous solid supports. Therefore, chemical and material engineers have made several strides to design and synthesize heterogeneous catalysts with enhanced catalytic activity and selectivity. As supported heterogeneous catalysts are composed of two important segments: (i) catalytically active metal nanoparticles (NPs), and (ii) support materials^18–20^, the developed methodologies to enhance the catalytic activity of metal NPs entails tailoring their morphologies, facets, and atomic arrangements^21–24^. Support materials have also been extensively developed to uncover facile recyclability, good physiochemical stability, high surface area for the accessibility of metal nanocatalysts to various substrates and reactants^25–28^. In addition, various approaches have been explored to functionalize the engineered heterogeneous catalysts thus enabling them to accommodate high loading capacity of catalytically active nanocatalysts and ameliorating their performance^29–34^.

For the next generation of heterogeneous catalysts endowed with specific functions and high catalytic activity, it is indispensable to design the architecture of engineered catalytically active NPs to be deposited in the accessible positions. Undeniably, the catalytic systems often developed by wet chemistry, the widely used method to prepare heterogeneous catalysts, cannot immobilize multiple catalytically active NPs on supports in a controllable manner at the designed nanoscale^35–37^. Even if endeavored, it requires very complicated and time-consuming multi-step process and the deployment of toxic chemical reagents. Therefore, new methods of designing and fabricating the heterogeneous catalysts comprising unique frameworks with various components should be planned.

Physically controlled decoration of catalytically active species on certain well-known and defined supports provides a great potential to fulfill this objective. Compared to the conventional wet-chemical methods, this strategy presents advantages of low-cost, and well-tolerated loading amounts and decoration locations on supports, which is unattainable by existing synthetic techniques. Consequently, we established in our approach the highly tolerable design of benign hosts and catalytically active species^38^.

A novel template-free and designable bottom-up grown Pd NPs deposited on vertically aligned SiO_2_ nanorod arrays (v-SiO_2_ NRs@Pd) nanostructured catalyst (prepared by glancing angle deposition (GLAD) method) is reported for the reduction of nitroarenes. Due to shadowing effect during the GLAD process, SiO_2_ NRs can be deposited on silicon wafers. As SiO_2_ NRs have open top structure, accessible Pd NPs can be precisely decorated on the surface of SiO_2_ NRs in a layer-by-layer architecture benefitting from a multiple-step GLAD method. Furthermore, our strategy enables the combinations of various metal NPs and support materials, and nano-scale controlled affixation of catalyst NPs robustly on nanorod arrays.

## Materials and methods

Oxide grains (SiO_2_ and SnO_2_) and metal pellets (Pd and Au) with 99.99% purity, used for film deposition, were purchased from Kojundo Chemistry. The other chemicals used in this study were purchased from the Daejung and Samchun Chemical Companies.

Fabrication of the v-SiO_2_ NRs@Pd nanostructured catalyst: A *p*-type Si wafers were cleaned in acetone, isopropanol, and deionized water under ultrasonication followed by drying in a nitrogen atmosphere. To fabricate SiO_2_ NRs, the evaporation was performed at a glancing angle with a rotation speed of 80 rpm; the wafer was located 50 cm away from the crucible, tilted at 80°. The base pressure was maintained at 1.0 × 10^–6^ Torr and growth rate was 1.0 Å s^−1^. After 250 nm-thick SiO_2_ NRs deposition, 3 nm-thick Pd film was sequentially deposited on the wafer at the original position (0°). These processes were repeatedly carried out until the 1 μm-thick v-SiO_2_ NRs were obtained. All the fabricated catalysts were annealed at 300 °C for 30 min in a reducing atmosphere to improves the crystallinity of Pd NPs and infixed them on the support promoting their catalytic activity and durability for recycling.

The morphology of the v-SiO_2_ NRs@Pd nanostructured catalyst was characterized using a field-emission scanning electron microscopy (FESEM, Zeiss SUPRA 55VP). The transmission electron microscopy (TEM) and cross-sectional high-resolution TEM images were obtained using a 200 kV filed-emission TEM (JEM-2100 F, JEOL). A focused ion beam (FIB, Helios 650) was utilized to prepare the samples for TEM analysis. The energy-dispersive X-ray spectroscopy (EDS) analysis was conducted to investigate the chemical mapping of the v-SiO_2_ NRs@Pd nanostructured catalyst. Grazing-incidence X-ray powder diffraction (GIXRD) was performed using PANalytical X’pert Pro X-ray diffractometer. The X-ray photoelectron spectroscopy (XPS) was carried out using AXIS Ultra DLD instrument (Kratos, U.K.) operating at a base pressure of 1.6 × 10^–10^ mbar at 300 K. An inductively coupled plasma atomic emission spectrometer (ICP-AES, Varian 730ES) was used for the measurement of Pd amounts. The products from the catalytic reactions were analyzed by a gas chromatography mass spectrometer (GC-MS) using an Agilent Technologies 7693 Autosampler. The structures of the chemical products were identified using a nuclear magnetic resonance (NMR, 600 MHz, High Resolution NMR Spectrometer, AVANCE Bruker).

## Results and discussion

**Figure 1:**
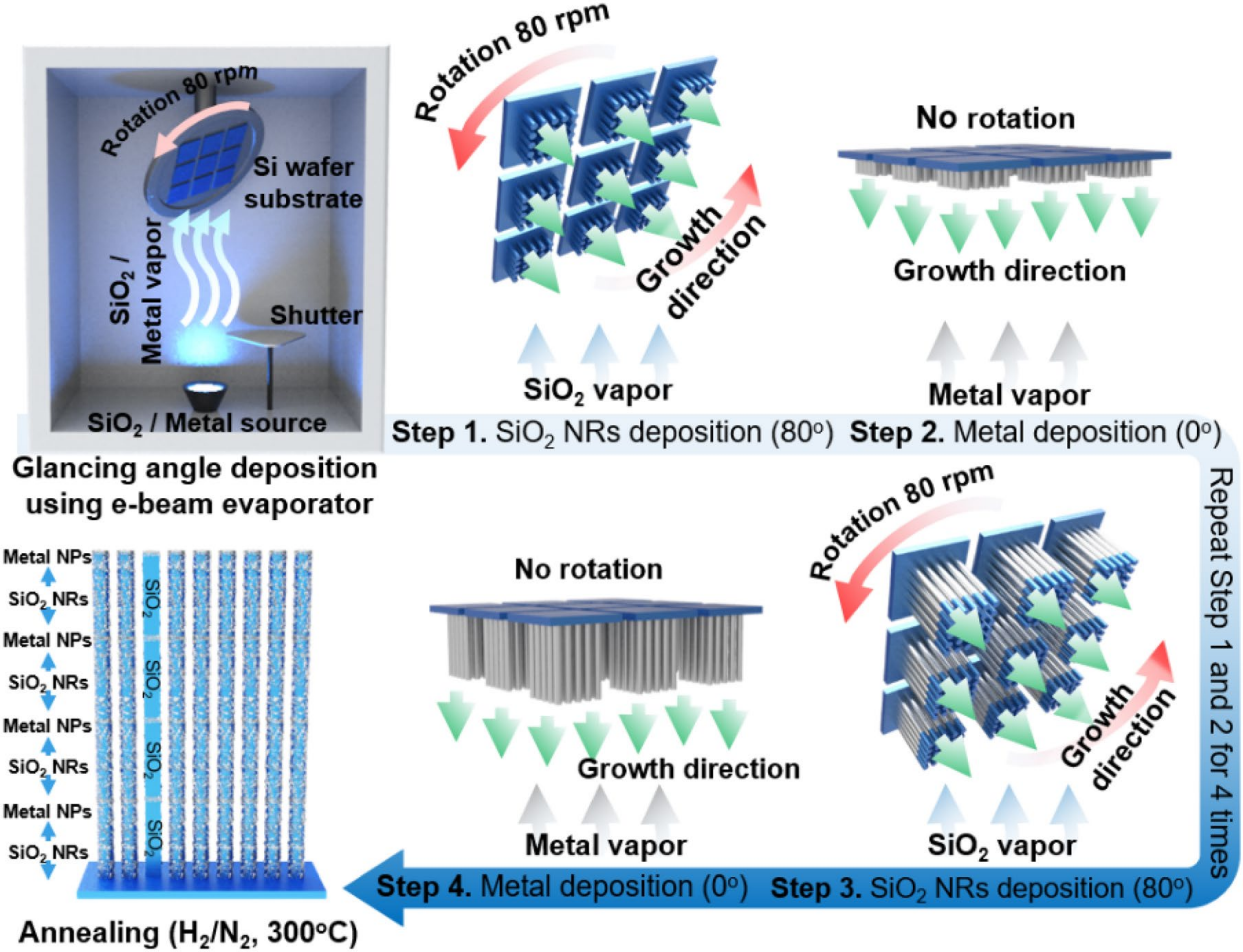
Synthetic procedure of v-SiO_2_ NRs@Pd nanostructured catalyst.

The fabrication process for the v-SiO_2_ NRs@Pd nanostructured catalyst using an electron beam evaporator is illustrated in Fig. 1. A multiple-step GLAD was utilized to deposit v-SiO_2_ NRs on a *p*-type Si wafer and effectively adorning Pd NPs on v-SiO_2_ NRs, thus substantially improving the previously reported methods^39–41^. SiO_2_ was deposited on a *p*-type Si wafer tilted at 80° and rotated with a speed of 80 rpm. After deposition of a 250 nm-thick layer of SiO_2_ NRs, a 3 nm-thick Pd layer was deposited at a tilt angle of 0° on the v-SiO_2_ NRs. These steps were performed repeatedly until the total length of the v-SiO_2_ NRs reached to 1 μm. Finally, the fabricated catalysts were annealed at 300 °C under a reducing atmosphere to furnish robustly stabilized Pd NPs.

**Figure 2:**
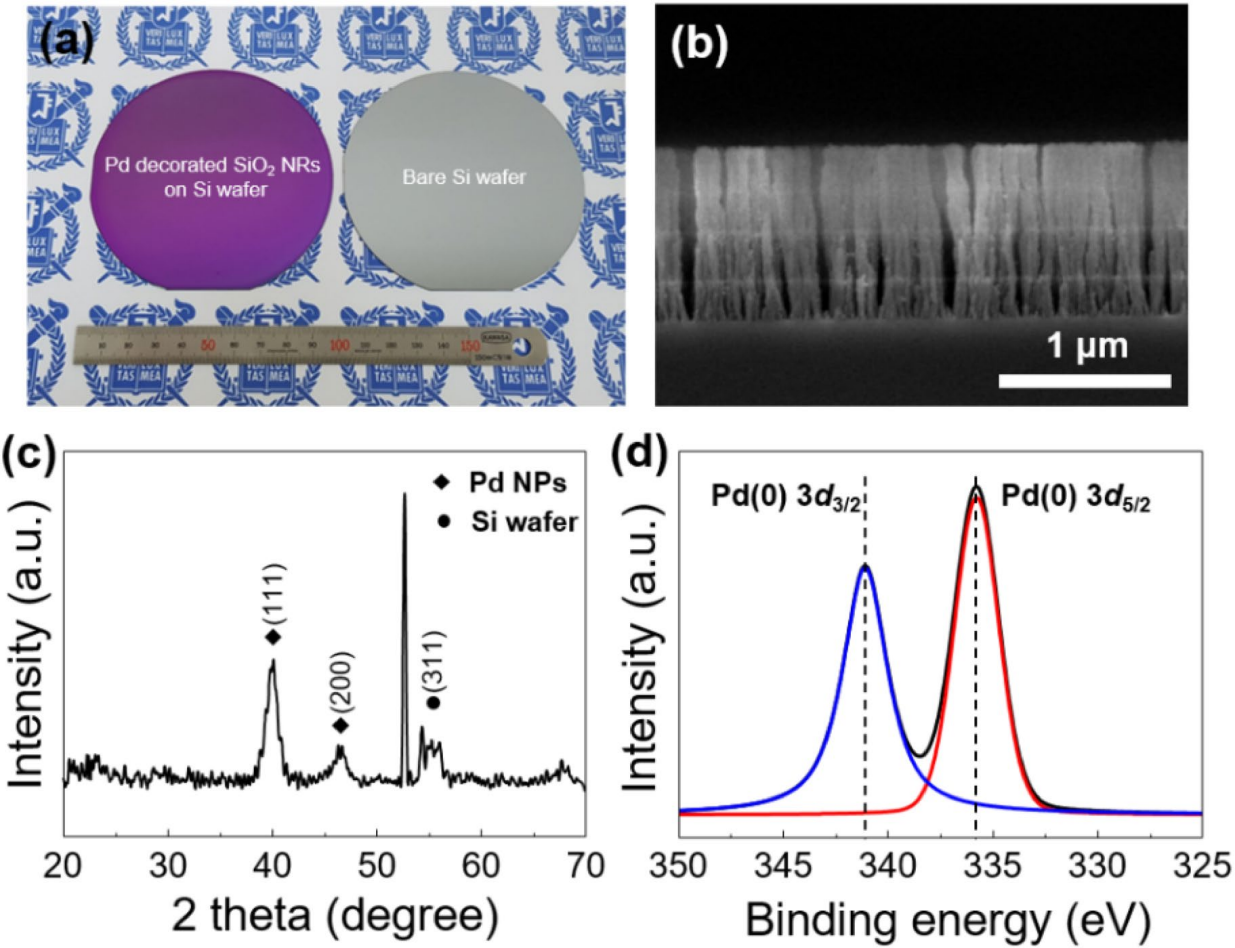
(**a**) A photographic image of v-SiO_2_ NRs@Pd nanostructured catalyst on p-type Si wafer (left) and bare p-type Si wafer. The diameter of the p-type Si wafers is 4 inches. (**b**) Cross-sectional SEM image of the v-SiO_2_ NRs@Pd nanostructured catalyst. (**c**) GIXRD pattern of v-SiO_2_ NRs@Pd nanostructured catalysts on *p*-Si wafer. (**d**) XPS core level spectrum of Pd 3d for v-SiO_2_ NRs@Pd nanostructured catalyst.

As shown in Fig. 2a and b, v-SiO_2_ NRs@Pd nanostructured catalyst was uniformly deposited on 4-inch Si wafer. The cross-sectional scanning electron microscopy image in Fig. 2b shows highly one-dimensional structures of v-SiO_2_ NRs@Pd catalyst on the Si wafer substrate. The nanorods were separated from each other by elongated pores and their average diameter was about 60 nm, improving the approachability of the Pd NPs and diffusion of the reactants and products. Due to their accessible nanostructure, the reagents can efficiently permeate into and interact with the v-SiO_2_ NRs@Pd nanostructured catalyst. To clarify the existence of Pd NPs in the v-SiO_2_ NRs, GIXRD was performed which revealed the peaks assignable to the (111) and (200) reflections of the face-centered cubic (FCC) Pd NPs (Fig. 2c). XPS analysis was also carried out to confirm the oxidation state of Pd species in v-SiO_2_ NRs@Pd nanostructured catalyst (Fig. 2d). The peaks corresponding to Si, O, and Pd are clearly observed in XPS spectra (Fig. S1). The Pd 3d_5/2_ and Pd 3d_3/2_ binding energies were found to be at 335.8 and 341.1 eV, respectively thus indicating the formation of zero-valent Pd species in v-SiO_2_ NRs@Pd nanostructured catalyst.

**Figure 3:**
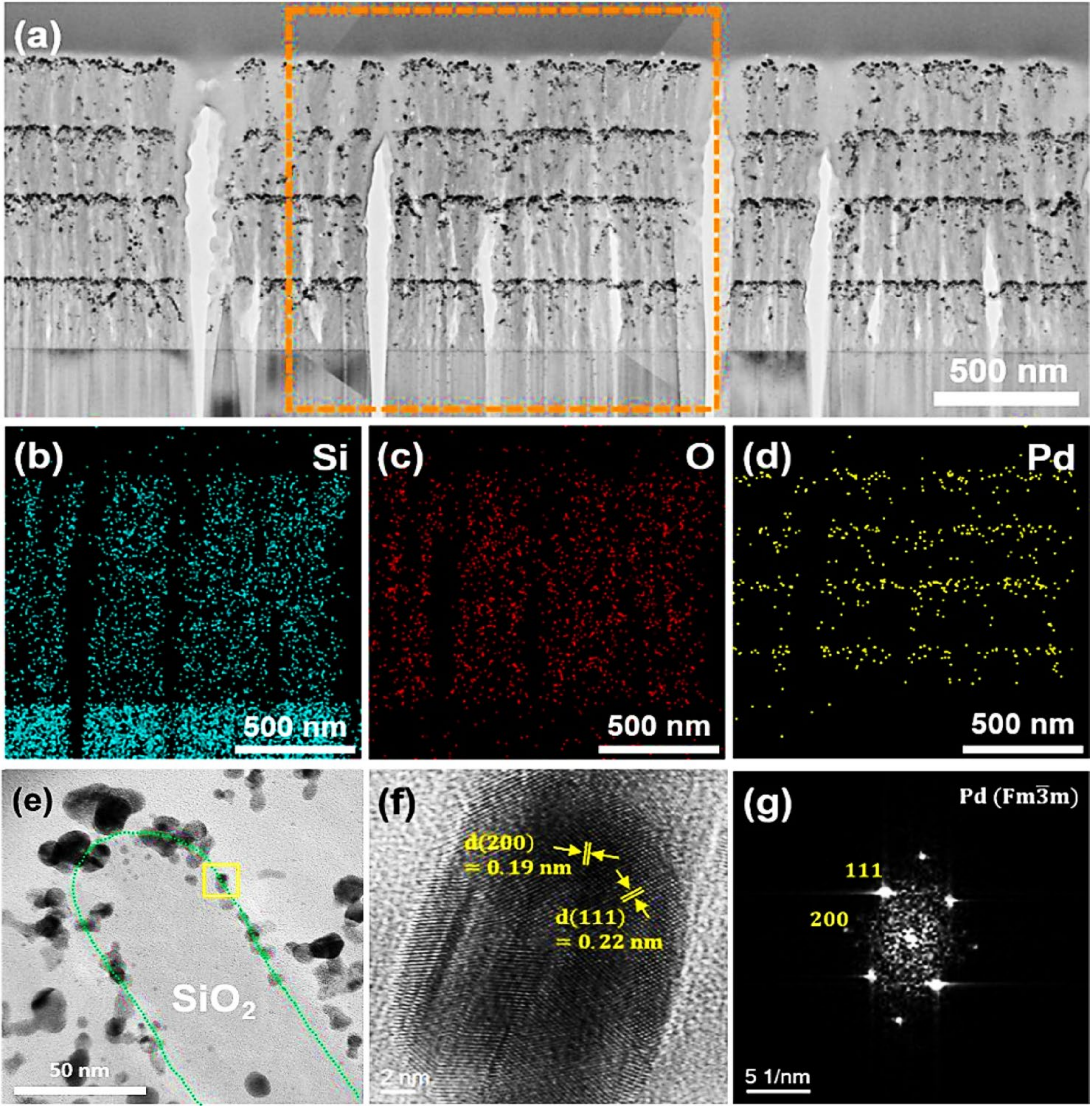
(**a**) Cross-sectional TEM image of the v-SiO_2_ NRs@Pd nanostructured catalyst. (**b**–**g**) EDS element maps of (**b**) Si, (**c**) O, and (**d**) Pd for the v-SiO2 NRs@Pd nanostructured catalyst obtained from the orange dashed rectangle of (**a**). (**e**) HRTEM images of v-SiO_2_ NRs@Pd nanostructured catalyst. (**f**) HRTEM images and (**g**) corresponding fast Fourier transform image of a Pd NP on SiO_2_ NRs obtained from the yellow rectangle in (**e**).

To further analyze the nanostructure of v-SiO_2_ NRs@Pd catalyst, TEM images were obtained as shown in Fig. 3a; four dense layers of Pd NPs were seen clearly deposited on v-SiO_2_ NRs. The Pd NPs were also observed in the side walls of the v-SiO_2_ NRs. The energy dispersive spectroscopy (EDS) mapping on the v-SiO_2_ NRs@Pd nanostructured catalyst was conducted. Figure 3b–d show the chemical mapping images of Si, O, and Pd elements for the selected areas in Fig. 3a (orange dashed rectangle). For Si and O elements, a uniform distribution across the v-SiO_2_ NRs has been observed. However, Pd NPs were distributed in a layer-by-layer architecture with total of four layers. The Pd element observed in between the layers has been attributed to the Pd NPs on sidewalls of v-SiO_2_ NRs. Figure 3e–g shows the high resolution TEM (HRTEM) images of Pd NPs on the surface (yellow rectangle) of the SiO_2_ NRs; Pd NPs were crystallized with the (111) preferred orientation. From the above results, it can be concluded that Pd NPs are efficiently decorated on the whole surface area of SiO_2_ NRs and immobilized at desired locations on SiO_2_ NRs through multiple-step GLAD method to realize a favorable spatial confinement. In other words, the catalytic active sites, Pd NPs, are well-refined on the v-SiO_2_ NR supports, resulting in more accessible catalytically activite sites on the v-SiO_2_ NRs@Pd nanostructured catalyst.

To further demonstrate the advantages of this physical architecture design, we fabricated v-SiO_2_ NRs decorated with Au and Pd alternately (v-SiO_2_ NRs@Pd/Au), using the same protocol. The four dense layers of metal NPs were observed on the v-SiO_2_ NRs@Pd/Au nanostructured catalyst (Fig. S2). The EDS mapping on the v-SiO_2_ NRs@Pd/Au nanostructured catalyst revealed that the Pd and Au NPs were layer-by-layer deposited on v-SiO_2_ NRs by turns. The HRTEM images also confirmed that the metal NPs were single crystalline in each layer for both, the Au and Pd NPs.

To confirm the oxidation state of Pd and Au NPs, XPS analysis was conducted. The peaks observed at 84.1, 87.8, 335.8, and 341.2 eV corresponded to Au 4*f*_7/2_, Au 4*f*_5/2_, Pd 3*d*_5/2_ and Pd 3*d*_3/2_, respectively. This indicated that the Pd and Au NPs on the v-SiO_2_ NRs@Pd/Au nanostructured catalyst were in zero-valent state. The similar catalyst was also synthesized on SnO_2_ supports using our strategy to broaden various type of supporting materials. As shown in Fig. S3, Pd NPs were well-decorated on v-SnO_2_ NRs. These results illustrated that metal NPs could be stabilized on the designed sites on various supports using the multiple-step GLAD physical method.

Compared with previously reported fabrication processes for nanostructured catalysts, this approach has several advantages including favorable design, precise architecture engineering, and accessible metal nanocatalysts for the reactants, etc.^42–44^*.* Using wet chemical methods, mainly applied for nanostructured catalysts fabrication, catalytic sites are randomly distributed due to the lack of controllability and the need for complicated functionalization processes^45^. In addition, it is very difficult to combine two or more metal NPs into the nanostructured support. However, this approach circumvents the limitations of previous reports for the catalysts on silicon wafers as the supporting materials via top-down fabrication process which often is accompanied by harmful and toxic HF etching^46^. The present strategy provides the varying catalytically active sites precisely integrated on the support and their location can be controlled on nanoscale, thus improving the approachability of the catalytic NPs to reactants.

To verify the catalytic activity and chemoselectivity of the v-SiO_2_ NRs@Pd nanostructured catalyst, we conducted the hydrogenation of various substituted nitroaromatics at room temperature; a reaction that has important impact in many industries, including environmental safety^47–50^. Our designed nanostructured catalyst presented superb catalytic activity for the selective hydrogenation of nitroaromatics in aqueous solution (Table 1). The procedure was accomplished under eco-friendly mild conditions, attaining high yields for the hydrogenation of substituted nitroaromatics to the corresponding aminoaromatics. The hydrogenation of nitroaromatics bearing two reducible groups could be achieved selectively to the amino compound as the only product, while another reducible group was retained unchanged (Table 1, entries 4–6). In addition, achieving highly selective reduction of nitro groups of aromatic molecules possessing olefin bonds is very challenging as there is high probability for both the functional groups to be readily hydrogenated. Importantly, this process proceeded with high selectivity (99%), reducing only nitro group (Table 1, entry 8) as affirmed by nuclear magnetic resonance spectroscopy (Fig. S4). Furthermore, a comparison of the catalytic performance study between the v-SiO_2_ NRs@Pd nanostructured catalyst and previously reported noble metal-comprising catalysts shows a highly comparable catalytic activity (Table S1).

**Table 1:** Heterogeneous reduction of substituted nitroarenes catalyzed by v-SiO_2_ NRs@Pd nanostructured catalyst.


Entry	Substrate	Product	Yields (%)^a^
1			99
2			92
3			93
4			91
5			94
6			92
7			95
8			94

Reaction conditions: Substituted nitroarenes (0.1 mmol), NaBH_4_ (0.12 mmol), v-SiO_2_ NRs@Pd nanostructured catalyst (1 mol% Pd), H_2_O (20 mL), room temperature, and 1.5 h. ^a^ Yields were determined by GC–MS.

To clarify the correlation between accessibility of Pd nanocatalysts and their activity, as a proof-of-concept, control experiments were carried out using various nanostructured catalysts in the hydrogenation of nitrobenzene as a model reaction under identical reaction conditions (Table S2). Initially, only Pd NPs were deposited on silicon wafer by GLAD method aiming for simple recycling though dipping and removing of the silicon substrate possessing stable nanocatalysts. The reaction produced a low yield of (15%) of aniline under optimized reaction conditions. FESEM images of this catalyst showed that the surface of the silicon wafer substrate was thoroughly covered by stacked Pd NPs without approachable opening for the diffusion of the reactants, resulting in low catalytic activity (Fig. S5). In addition, micro- and nano-space channels provide permeable paths to organic reagents, thus improving the accessibility of the Pd nanocatalysts^51–53^. We also fabricated and evaluated the catalytic activity of Pd NPs on SiO_2_ arrays having ~ 200 nm height (Fig. S6). Interestingly, the SiO_2_ columns exposing Pd NPs could hydrogenate the nitro group of nitrobenzene in relatively higher yields (35%). Although the prototype short length SiO_2_ arrays were partly aggregated, from this result we found that the Pd NPs stabilized on sturdy SiO_2_ support functioned to some extent effectively, which can be attributed to internal surface and pore diffusivity^54^. Consequently, we designed and fabricated SiO_2_ arrays and aligning them separately from each other with ~ 600 nm length possessing Pd NPs on their scaffolds as depicted in FESEM image (Fig. S7). Although the Pd NPs were located irregularly on the SiO_2_ arrays, their combination improved their interactions with the solvent and reactants via the accessible pores, promoting efficient catalytic reactions (Table S1, entry 3). Finally, we designed and developed fully arranged and assorted v-SiO_2_ NRs@Pd catalyst having permeable tunnel paths and elongated pores between SiO_2_ columns with four strata of attainable Pd NPs, and maximizing their catalytic activity in the given geometric configuration. Similarly, the alternative four dense layers of Pd NPs and Au NPs in the v-SiO_2_ NRs@Pd/Au nanostructured catalyst provided high catalytic activity in the model reduction. The combination and the activity of different nanocatalysts can easily be tuned and optimized by this method, controlling their locations on supports and distance between the nanocatalysts and the arrays. The controlled pores in the nanostructure provided facile adsorption of reactants to the Pd NPs and subsequent desorption of products from them. Additionally, a larger amount of Pd NPs can be decorated on the whole surface of SiO_2_ NRs exposing large amount of accessible Pd sites for the catalytic reactions. Thus, v-SiO_2_ NRs@Pd nanostructured catalyst could expedite Suzuki coupling reaction of various aryl halides with phenylboronic acid (Table 2); reaction of aryliodides and arylbromides with phenylboronic acid proceeded with excellent conversion in 3 h and 5 h, respectively.

**Table 2:** Suzuki cross–coupling reactions catalyzed by v-SiO_2_ NRs@Pd nanostructured catalyst.


Entry	Substrate	Time (h)	Yields (%)^a^
1		3	97
2		3	94
3		5	95
4		5	91
5		5	87

Reaction conditions: Aryl halide (0.1 mmol), phenylboronic acid (0.12 eq.), v-SiO_2_ NRs@Pd nanostructured catalyst (1 mol%), K_2_CO_3_ (1.5 eq.), DMF/H_2_O (5:1), 100 °C. ^a^Yields were determined by GC–MS.

Recycling and reuse of the catalysts are crucial parameters because of the environmental impact and practical applications. Although many heterogeneous catalysts have been developed for the reduction of nitroaromatics, they generally entail very arduous recycling procedures such as centrifugation, filtration, laborious work-up purification, etc. However, v-SiO_2_ NRs@Pd nanostructured catalyst could be easily separated being withdrawn from the reaction mixture by tweezers, as they have a monolithic structure, a salient advantage of our catalyst. The durability and reusability of our catalyst was investigated for the hydrogenation of nitrobenzene. After completion of the reaction, the v-SiO_2_ NRs@Pd nanostructured catalyst was simply removed from the reaction mixture, rinsed with water, and reused in the next cycle. The activity of the catalyst remained stable even after seven consecutive reaction cycles, though there was a minuscule decline in activity of the catalyst (Table 3). As the catalytic activity of v-SiO_2_ NRs@Pd nanostructured catalyst originates from its special architecture engineering of one body nanostructure, we precisely characterized the recovered v-SiO_2_ NRs@Pd nanostructured catalyst by EDX, TEM, and HRTEM techniques (Fig. S8), affirming the constant stability of the designed catalyst after the recycling. Consequently, Pd leaching could be the reason for this catalytic decrease, which was supported by ICP-AES analysis; negligible Pd species was detected.

**Table 3:**
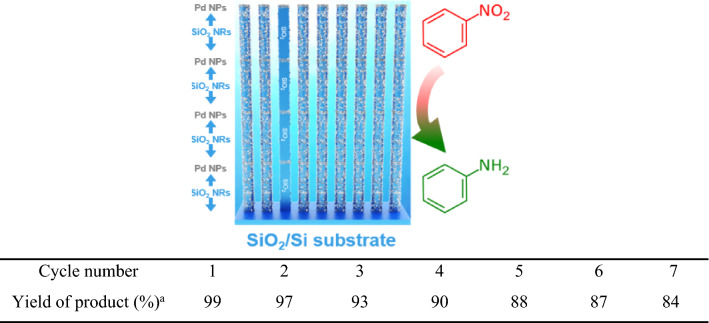
Reuse of v-SiO_2_ NRs@Pd nanostructured catalyst in the heterogeneous reduction of nitrobenzene.

Reaction conditions: Substituted nitroarenes (0.1 mmol), NaBH_4_ (0.12 mmol), v-SiO_2_ NRs@Pd nanostructured catalyst (1 mol% Pd), H_2_O (20 mL), room temperature, and 1.5 h.

^a^Yields were determined by GC–MS.

## Conclusions

The novel synthesis of v-SiO_2_ NRs@Pd nanostructured catalyst described in this study exhibited promising catalytic characteristics for the hydrogenation of nitroaromatics and the Suzuki cross–coupling reaction. The vertically aligned SiO_2_ NRs provided an excellent platform for Pd nanocatalysts to be firmly cohered to create a monolithic structure, which is superior in the realization of a simple recycling process. Because this fabrication procedure is based on physical vapor deposition under vacuum conditions, it can be applied to various combinations of metal nanocatalysts and supporting nanostructures without complicated synthesis procedures often deployed in wet chemical methods. The effective design of nano-architectures for a monolithic nanocatalysts-attached SiO_2_ NRs will expectedly open new perspectives toward the development of highly recyclable nanocatalysts for other catalytic reactions.

## Supplementary Information

Supplementary Information.
