# Supplementary material for: Architecture engineering of nanostructured catalyst via layer-by-layer adornment of multiple nanocatalysts on silica nanorod arrays for hydrogenation of nitroarenes
Source: Sci Rep. 2022 Jan 6;12:2. doi: 10.1038/s41598-021-02312-0 (PMC8738731; doi:10.1038/s41598-021-02312-0)
Supplement: Supplementary file 1 — Supplementary Information. [file 41598_2021_2312_MOESM1_ESM.docx]

**Supporting information**

**Architecture engineering of nanostructured catalyst via layer-by-layer adornment of multiple nanocatalysts on silica nanorod arrays for hydrogenation of nitroarenes**

Kootak Hong^1,+^, Jun Min Suh^1,+^, Tae Hyung Lee^1^, Sung Hwan Cho^1^, Seeram Ramakrishna^2^, Rajender S. Varma^3,^*, Ho Won Jang^1,^*, and Mohammadreza Shokouhimehr^1,^*

^1^Department of Materials Science and Engineering, Research Institute of Advanced Materials, Seoul National University, Seoul 08826, Republic of Korea.

^2^Center for Nanotechnology and Sustainability, Department of Mechanical Engineering, National University of Singapore, 119260, Singapore.

^3^Regional Centre of Advanced Technologies and Materials, Palacký University, Šlechtitelů 27, 783 71 Olomouc, Czech Republic.

Corresponding Authors

varma.rajender@epa.gov (R.S.V.), hwjang@snu.ac.kr (H.W.J.), mrsh2@snu.ac.kr (M.S.)

+ K.H., J.M.S., contributed equally to this work.

**Heterogeneous reduction of substituted nitroarenes**

The reduction of nitroarenes was performed in a glass flask. In a typical procedure, v-SiO_2_ NRs@Pd nanostructured catalyst (1 mol%) was immersed in H_2_O (20 mL). Then, a nitroarenes compound (0.1 mmol), NaBH_4_ (0.12 mol%) and a small stirring bar were added to the glass flask. The reaction mixture was stirred at room temperature for 1.5 h under air atmosphere. After completion of the reaction, the v-SiO_2_ NRs@Pd nanostructured catalyst was drawn out from the reaction mixture by tweezers, rinsed with ethanol, and reused in the next cycle.

**Heterogeneous Suzuki cross-coupling reactions**

Suzuki reactions were expedited using v-SiO_2_ NRs@Pd nanostructured catalyst in a round-bottom flask containing dimethylformamide (DMF)/H2O (5:1) solvent. Aryl halide (0.1 mmol), phenylboronic acid (0.12 eq.), K_2_CO_3_ (1.5 eq.), and a small stirring bar were added to the flask immersed in an oil bath (100 °C). The reaction mixtures were stirred under air atmosphere for adequate time to obtain the expected biphenyl products.


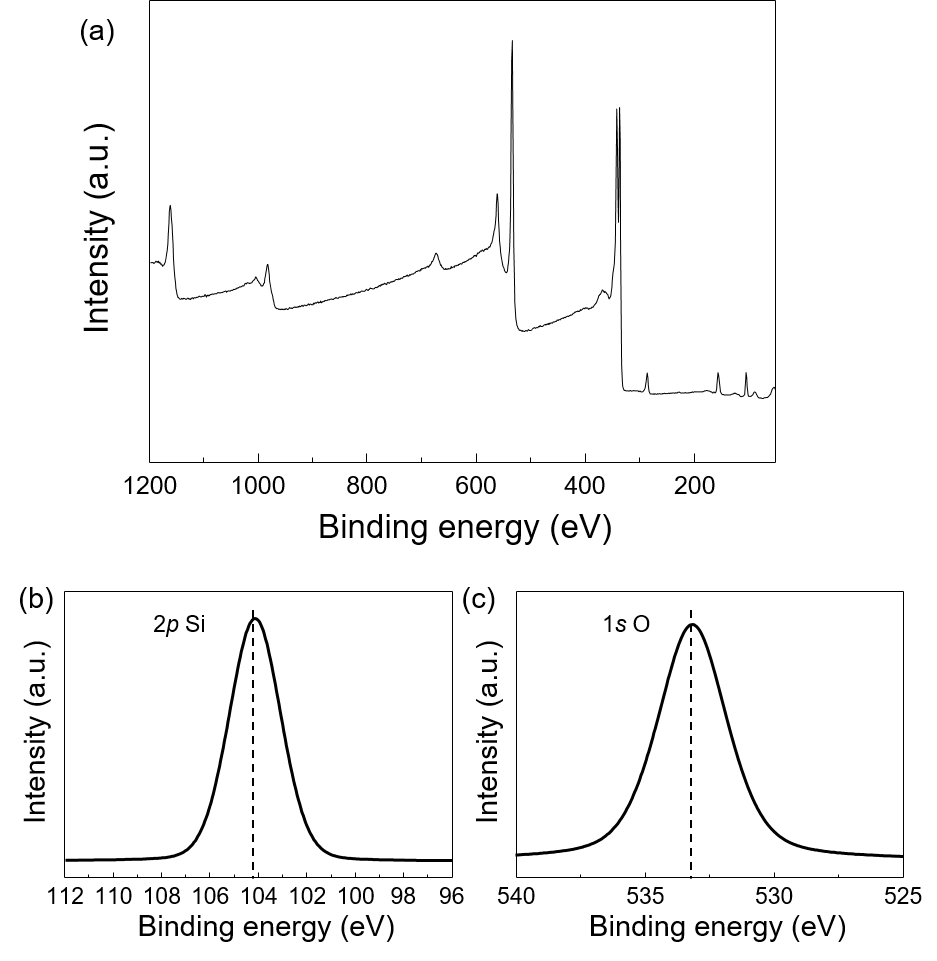


**Fig. S1.** XPS analysis of (a) survey, (b) Si, and (c) O present in v-SiO2 NRs@Pd

nanostructured catalyst.


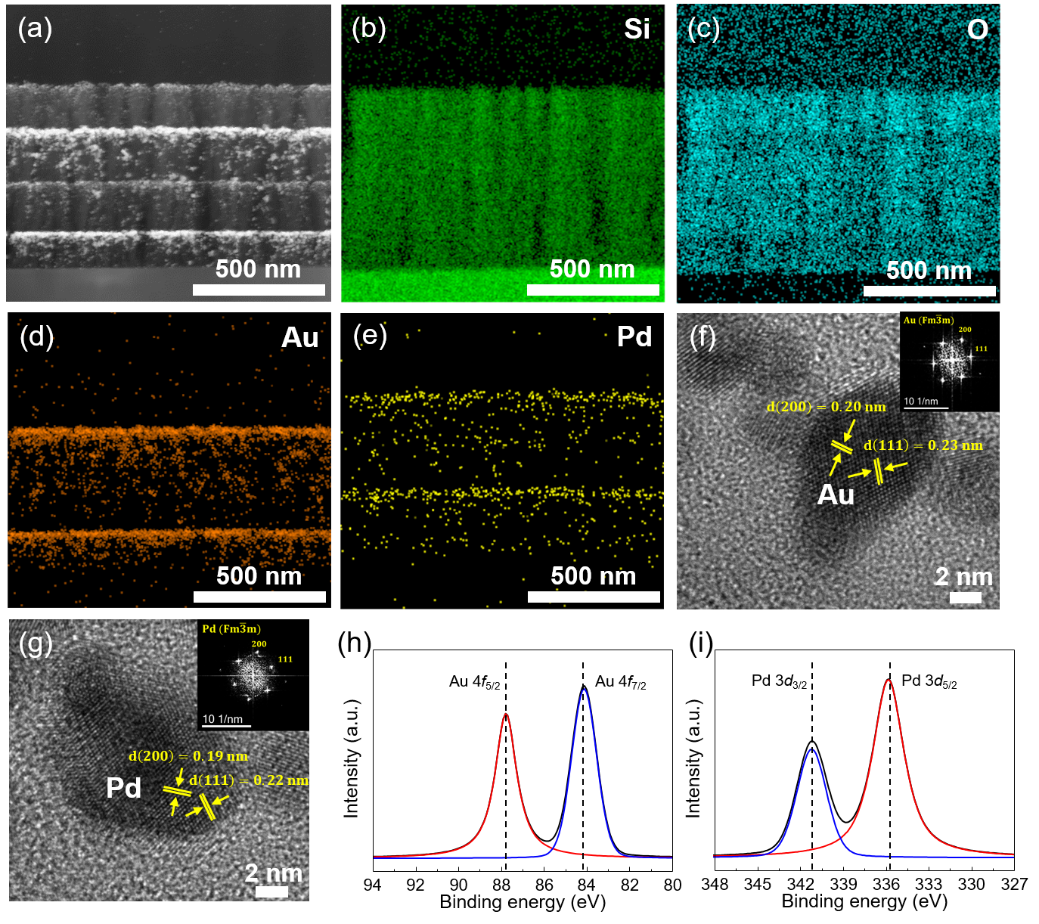


**Fig. S2.** (a) Cross-sectional FESEM image of v-SiO_2_ NRs@Pd/Au nanostructured catalyst. (b-e) EDS element maps of (b) Si, (c) O, (d) Au, and (e) Pd of v-SiO_2_ NRs@Pd/Au nanostructured catalyst. HRTEM images of (f) Au and (g) Pd existing in v-SiO_2_ NRs@Pd/Au nanostructured catalyst. XPS analysis of (h) Au and (i) Pd in v-SiO_2_ NRs@Pd/Au nanostructured catalyst.


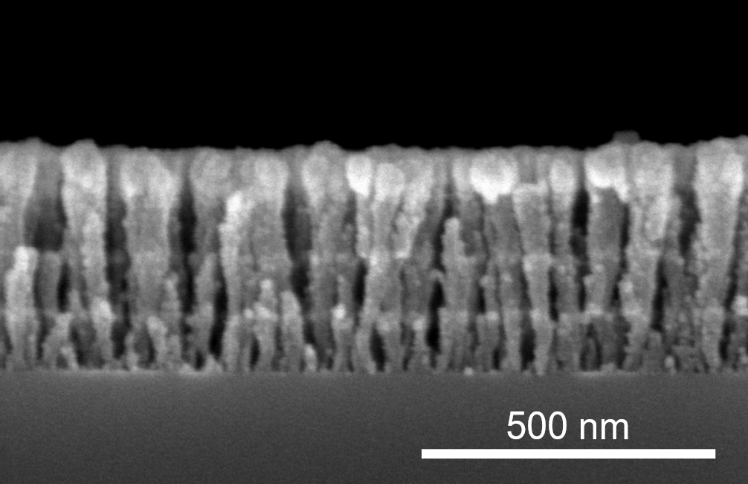


**Fig. S3.** Cross-sectional FESEM image of v-SnO_2_ NRs@Pd nanostructured catalyst.

**Fig. S4.** C-13 NMR spectrum of 3-vinylbenzenamine (Table 1, entry).

**
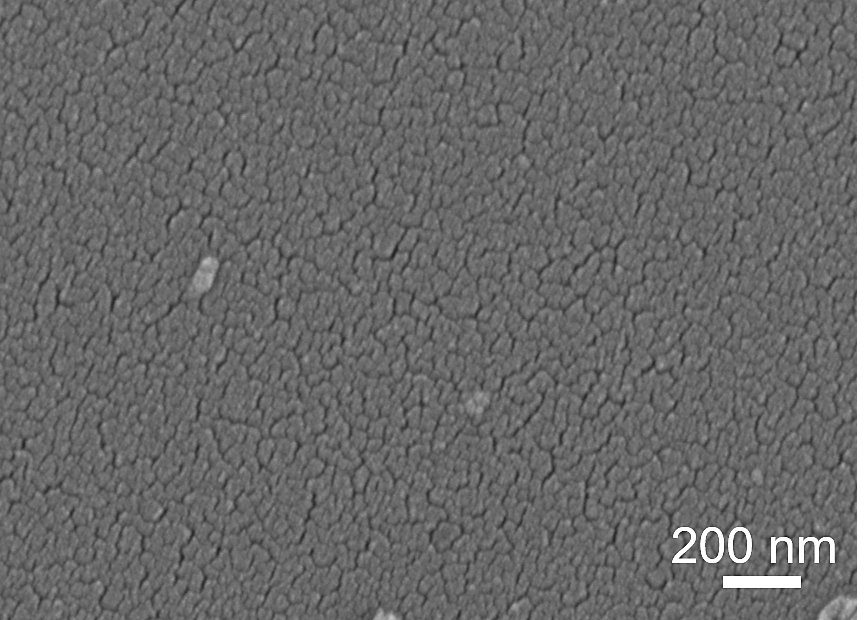
**

**Fig. S5.** FESEM image of Pd NPs deposited directly on silicon wafer.

**
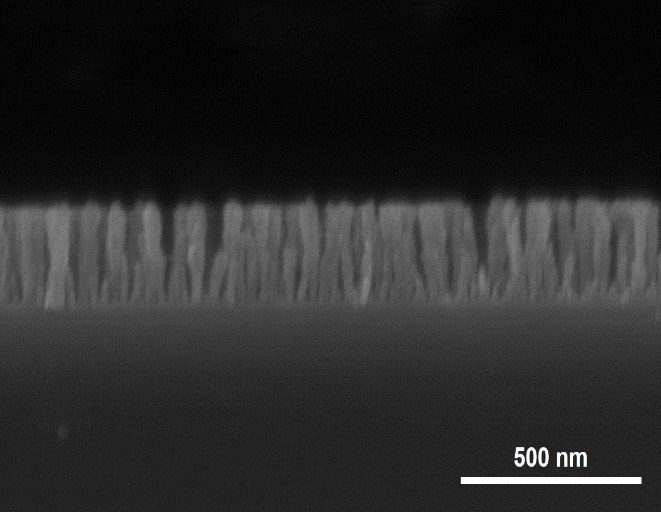
**

**Fig. S6.** Cross-sectional FESEM image of ~200 nm v-SiO_2_ NRs@Pd nanostructured catalyst.


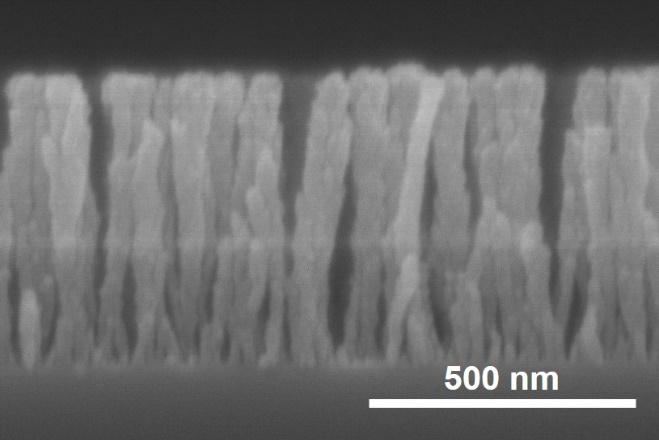


**Fig. S7.** Cross-sectional FESEM image of ~600 nm v-SiO_2_ NRs@Pd nanostructured catalyst.


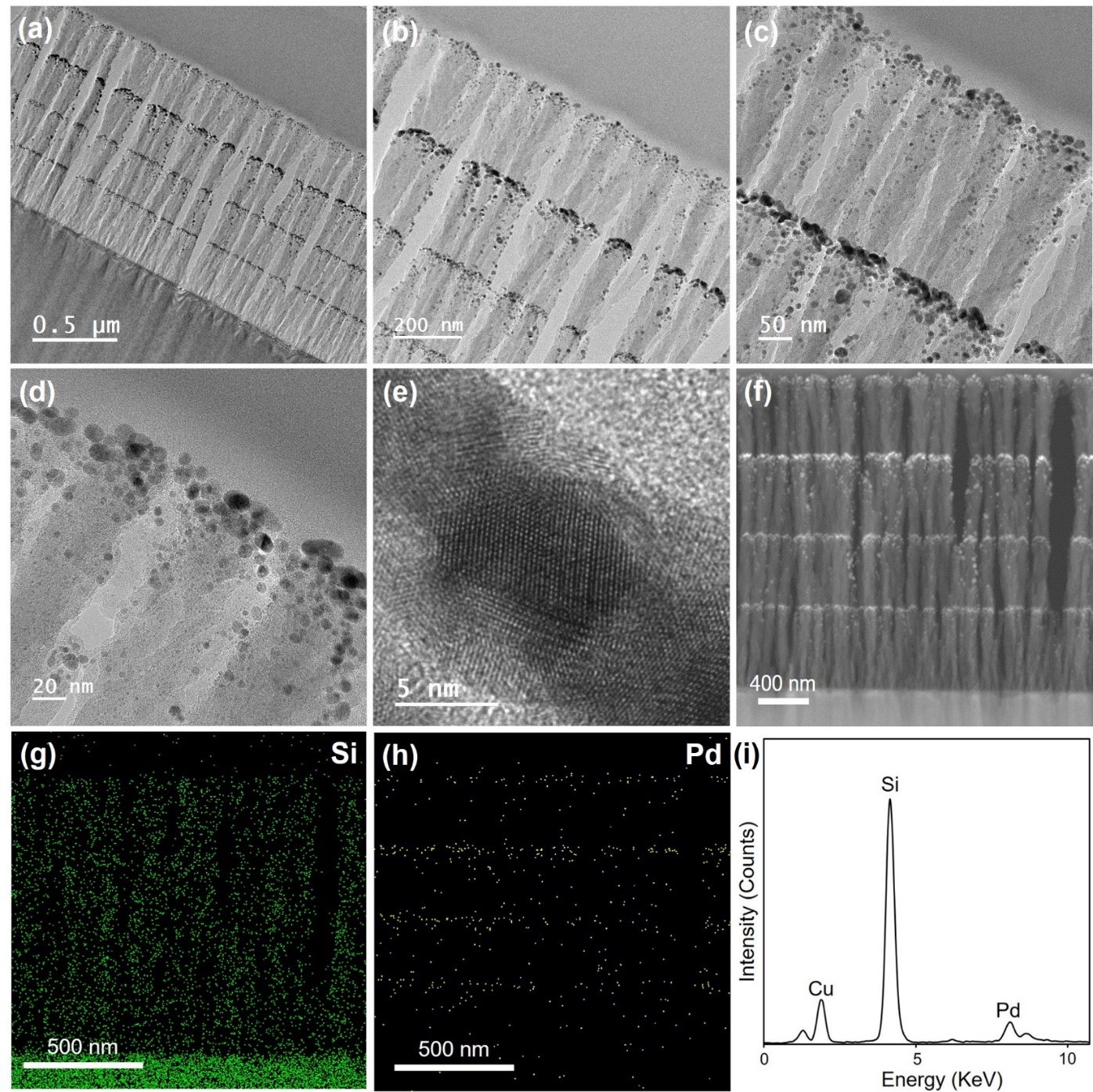


**Fig. S8.** (a-e) Cross-sectional TEM and HRTEM images, and (F) STEM image of recycled v-SiO_2_ NRs@Pd nanostructured catalyst. (g) Si, and (h) Pd elemental mapping of recycled v-SiO_2_ NRs@Pd nanostructured catalyst. (i) EDX spectrum of recycled v-SiO_2_ NRs@Pd nanostructured catalyst.

**Table S1.** Comparative catalytic studies for the reduction of nitroaromatics.

| **Entry** | **Catalyst** | **Reaction conditions** | **Yield (%)** | **Refs.** |
| --- | --- | --- | --- | --- |
| 1 | Hollow nanocomposite Rh (1 mol%) | Hydrazine (1 mmol), EtOH, 80 ^o^C, 2.5 h | 91-99 | S1 |
| 2 | Magnetic carbon nanocomposite silica Pt (1 mol%) | Hydrazine (1 mmol), EtOH, 80 ^o^C, 3 h | 94-99 | S2 |
| 3 | Pd nanocatalysts confined in mesoporous silica (1 mol%) | Hydrazine (1 mmol), EtOH, 80 ^o^C, 4 h | 91-99 | S3 |
| 4 | Core-shell Pd-iron oxide nanocatalyst (1 mol%) | Hydrazine (1 mmol), EtOH, 80 ^o^C, 4 h | 92-99 | S4 |
| 5 | Ag nanocomposite (5 mol%) | NaBH_4_ (10 mmol), H_2_O, r.t., 0.5-6 h | 71-94 | S5 |
| 6 | Pd nanopartciles on cyclodextrin (0.5 mol%) | NaBH_4_ (3 mmol), H_2_O, r.t., 3 h | 93-99 | S6 |
| 7 | v-SiO_2_ NRs@Pd nanostructured catalyst (1 mol%) | NaBH_4_ (1.2 mmol), H_2_O, r.t., 1.5 h | 91-99 | This work |

**Table S2.** Heterogeneous reduction of nitrobenzene catalyzed by different nanostructured catalyst.

| **Entry** | **Nanostructured catalyst** | **Yields (%)^a^** |  |
| --- | --- | --- | --- |
| 1 | Pd NPs on silicon wafer | 15 |  |
| 2 | Pd NPs on ~200 nm SiO_2_ NRs | 35 |  |
| 3 | Pd NPs on ~600 nm SiO_2_ NRs | 58 |  |
| 4 | v-SiO_2_ NRs@Pd catalyst | 99 |  |

Reaction conditions: Nitrobenzene (0.1 mmol), NaBH_4_ (0.12 mmol), nanostructured catalyst (1 mol% Pd), H_2_O (20 mL), room temperature, and 1.5 h. ^a^ Yields were determined by GC-MS.

**References**

S1. M. Shokouhimehr, J. E. Lee, S. I. Han, T. Hyeon, *Chem*. *Commun*., 2013, **49**, 4779.

S2. M. Shokouhimehr, T. Kim, S. W. Jun, K. Shin, Y. Jang, B. H. Kim, T, Hyeon, *Appl*. *Catal*. *A*. *Gen*., 2014, **476**, 133.

S3. A. Kim, S. M. Rafiaei, S. Abolhosseini, M. Shokouhimehr, *Energy Environ*. *Focus*, 2015,

**4**, 18.

S4. M. Shokouhimehr, K. Y. Shin, J. S. Lee, M. J. Hackett, S. W. Jun, M. H. Oh, J. Jang, T.

Hyeon, *J*. *Mater*. *Chem*. *A*, 2014, **2**, 7593.

S5. S. Giri, R. Das, C. V. D. Westhuyzen, A. Maity, *Appl*. *Catal*. *B*. *Environ*., 2017, **209**, 669.

S6. Y. Guo, F. Zhao, G. Lan, L. Li, Y. Liu, Y. Si, Y. Jiang, B. Yang, R. Yang, *RSC Advances*,

2016, **6**, 7950.
